# Radiographically Negative, Asymptomatic, Sentinel Lymph Node Positive Cutaneous T-Cell Lymphoma in a 3-Year-Old Male: A Case Report

**DOI:** 10.1155/2012/791602

**Published:** 2012-10-23

**Authors:** Jeffrey Carson, Jiri Bedrnicek, Shahab Abdessalam

**Affiliations:** ^1^Department of General Surgery, University of Nebraska Medical Center, Omaha, NE 68198, USA; ^2^Department of Pathology, Children's Hospital & Medical Center, Omaha, NE 68114, USA; ^3^Department of Surgery, Children's Hospital & Medical Center, Omaha, NE 68114, USA

## Abstract

We present a case of a 3-year-old male originally diagnosed with a CD30+ anaplastic cutaneous T-cell lymphoma with no evidence of systemic disease after CT scan, PET scan, and bone marrow aspiration. Sentinel lymph node biopsy (SLNB) was performed as an additional step in the workup and showed microscopic disease. Current management/recommendations for cutaneous T-cell lymphoma do not include SLNB. Medical and surgical management of cutaneous malignancies is dramatically different for local versus advanced disease. Therefore adequate evaluation is necessary to properly stage patients for specific treatment. Such distinction in extent of disease suggests more extensive therapy including locoregional radiation and systemic chemotherapy versus local excision only. Two international case reports have described SLNB in cutaneous T-cell lymphoma with one demonstrating evidence of node positive microscopic disease despite a negative metastatic disease workup. This case is being presented as a novel case in a child with implications including lymphoscintigraphy and SLNB as a routine procedure for evaluation and staging of cutaneous T-cell lymphoma if the patient does not demonstrate evidence of metastatic disease on routine workup.

## 1. Introduction 

Cutaneous T-cell lymphomas (CTCLs) are a heterogeneous group of lymphoproliferative disorders characterized by infiltration of the skin by malignant T cells. Primary cutaneous CD30+ T-cell lymphoma is a rare subtype of CTCLs. Prognosis is favorable with a 5-year survival of 90% [1]. However, for those diagnosed with stage IV disease, median survival is less than three years [2]. Treatment for CTCL is dependent on subtype and extent of disease. Therapies vary from wide local excision with local topical treatment to systemic chemotherapy, radiation therapy, and allogeneic stem cell transplant. Therefore determining disease extent is of critical importance for prognostic value as well as proper management. Systemic evaluation with sonography of lymph node basins, CT scan, PET scan, and bone marrow aspiration has served to evaluate the extent of disease. Sentinel lymph node biopsy (SLNB) has not been used for routine evaluation of CTCLs however it has been used extensively for evaluation of multiple other cutaneous and noncutaneous malignancies. Krämer et al. published a case from Germany where they reported negative metastatic workup but microscopic evidence of disease on sentinel lymph node biopsy [3]. This modality is a potential diagnostic method, which if used routinely could identify higher stage disease on presentation than initially possible with current standards for diagnosing metastatic disease for cutaneous malignancies.

## 2. Case Presentation 

A 3-year-old boy, with no significant past medical history, initially presented with a “pinkish” lesion on his abdomen that was first thought to be a “bug bite.” When the spot did not resolve after six weeks, he was referred to a dermatologist who performed an excisional biopsy. He had no evidence of lymphadenopathy in any of his nodal basins on physical exam. 

**Figure 1:**
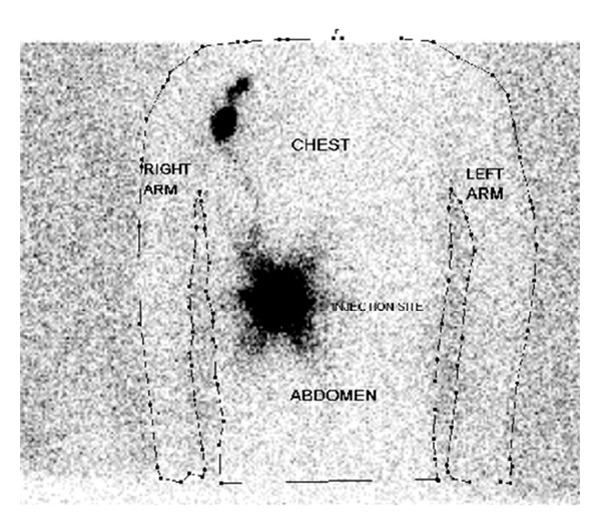
Lymphoscintigraphy demonstrating uptake in the right axilla.

**Figure 2:**
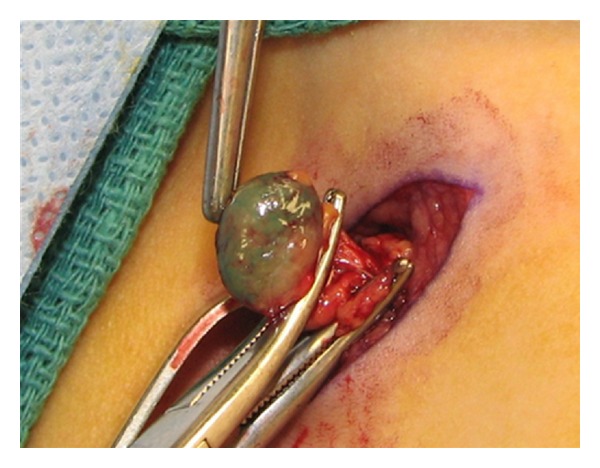
One of the “blue/hot” lymph nodes being removed from the right axilla.

**Figure 3:**
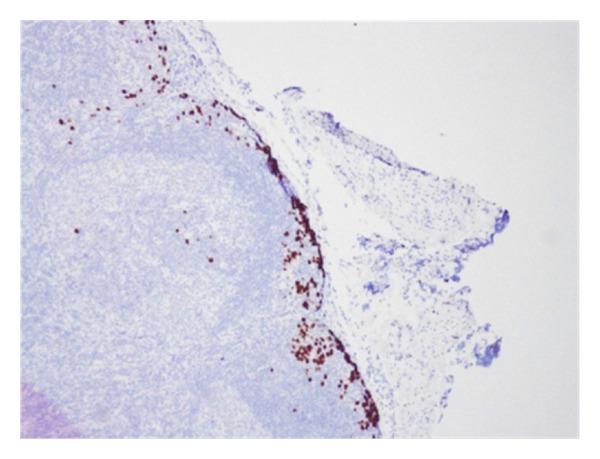
ALK-1 immunohistochemistry of sentinel lymph node demonstrating metastatic cells.

**Figure 4:**
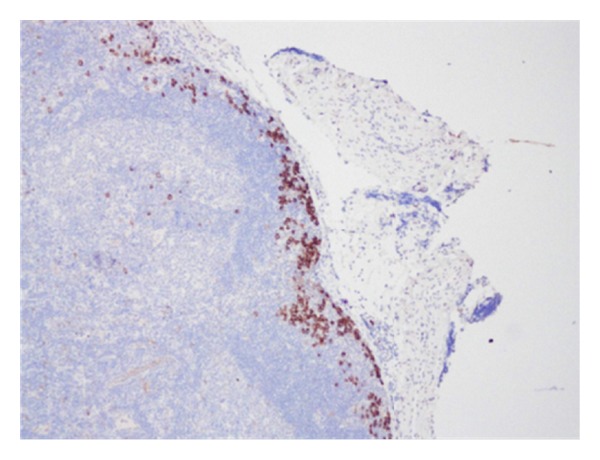
CD30 immunohistochemistry of sentinel node demonstrating metastatic cells.

Histopathology revealed an anaplastic T-cell lymphoma with negative, but close, margins. He underwent full-body PET and CT scans to look for any evidence of distant disease, and these studies were all negative. Further molecular and immunohistochemical workup demonstrated positivity for ALK (2p23) rearrangement as well as CD30+ cells. He was referred to pediatric surgery at this point for wide local excision of the previous biopsy site. In addition, it was proposed to also perform an SLNB. Lymphoscintigraphy, done preoperatively, using technetium 99 was performed and showed the draining nodes in his right axilla (see Figure 1). Lympozurin blue was injected around the previous biopsy site, and exploration of the right axilla revealed two sentinel nodes with greater than 4 times the background radiation that were blue. These were excised (see Figure 2). He also underwent bone marrow biopsy, which demonstrated no lymphoma population by histological examination, immunohistochemical staining or FISH cytogenetics. The excised sentinel lymph nodes were both positive for anaplastic large cell lymphoma, containing focal subcapsular involvement by anaplastic large cell lymphoma, which stained positive for ALK-1 and CD30 (see Figures 3 and 4). Wide local excision of original tumor site contained atypical lymphoid infiltrate, negative for CD30 nor ALK-1, in the dermis.

He was placed on POG 9219 receiving a combination of cyclophosphamide, vincristine, doxorubicin, and steroids over the next three months. He is now 8 months after completing all of his therapy and is without evidence of recurrent disease.

## 3. Discussion 

We present a unique case of a child with metastatic anaplastic CTCL whose metastatic workup was initially negative by conventional surveillance methods. CD30+ CTCL is a rare occurrence in patients younger than 20 years old and only constitutes 25% of all cases of CTCLs for all ages. This cutaneous neoplasm is characterized by erythematous or ulcerated solitary nodules, varying in size from 2 to 10 cm in diameter [4, 5]. Histologically, it is characterized by diffuse, nonepidermotropic infiltrates with cohesive sheets of large, mature CD30-positive tumor cells, oval or irregularly shaped nuclei, prominent eosinophilic nucleoli, and abundant cytoplasm. This presentation is consistent with our case both grossly and histologically. Additional common features are epidermal ulceration (63%), prominent vascular proliferation (60%), pseudoepitheliomatous hyperplasia (55%), tumor necrosis (55%), and vascular infiltration by neoplastic cells (44%) [6]. 

Lymphoscintigraphy and sentinel lymph node biopsy have been present in oncological evaluation of disease spread for decades and have been established as standard of care for staging disease extent for numerous malignancies such as breast cancer and melanoma [7, 8]. More recently lymphatic mapping and sentinel lymph node biopsy have emerged as a promising minimally invasive surgical technique to detect metastatic nodes in patients with malignancies other than melanoma and breast, including colon, esophageal, gastric, lung, head and neck, and thyroid cancers [9–11]. However, this method has not been routinely used in the evaluation of other cutaneous malignancies such as CTCLs. In addition, in the pediatric population, SLNB has not achieved the universal acceptance that it has with adults, with only a few pediatric centers across the country performing the procedure, and currently no COG (Children's Oncology Group) protocols using SLNB as the standard, but rather as an “option” [12]. We propose that children are not different, and if lymph node metastases are a possibility in pediatric malignancies with “standard” metastatic workup being negative, that SLNB should strongly be considered, as multiple studies in adults have confirmed its efficacy and accuracy.

Two case reports currently exist in the surgical literature describing use of sentinel lymph node biopsy for evaluation of CTCL. One of the two cases identified metastatic disease and subsequently underwent advanced treatment including chemotherapy and radiation therapy [3]. Both reports conclude that, due to significantly different prognoses as well as extent and mode of treatment for local versus metastatic disease, knowledge of lymph node status may be beneficial for management of CTCL [3, 13]. 

Interestingly each case report does not identify any specific reason or indication for undergoing sentinel lymph node biopsy. Rather, lymph node biopsy was done out of “intuition.” While this may not be standard practice in an evidence-based medicine era, it certainly has its merits when positive findings alter treatment plans. It very well may be the intuition of an experienced clinician that spurs change in management in such circumstances. While this case is the only one of its kind reported in the United States, it also highlights a unique event in the pediatric population. Treatment modalities and survival in this population may have a greater impact on quality of life and future health for the child. Therefore accurate diagnosis and proper treatment are critical in this population.

While there is no prospective randomized clinical trial currently available to evaluate the efficacy of SLNB in CTCL, there is evidence to suggest that it may play an important role in proper staging for this disease. Cases like the one presented here demonstrate the utility of proper staging and provide support for the idea of routinely using this modality as a diagnostic method. It has proven to be effective and is currently the gold standard for other malignancies with much higher incidence. Therefore we suggest its use more frequently for CTCLs and other malignancies where precise staging can lead to proper treatment and more accurate prognosis for patients.

## 4. Conclusion 

CTCLs are a unique dermatological malignancy with potential to have favorable prognosis if staged and treated accurately. The case presented of a CD30+ CTCL in a 3-year-old male is a rare event where treatment was altered by a diagnostic procedure well established for other cutaneous malignancies, but not frequently used for CTCL. Although this paper highlights only one case it should bring attention to the utility of a low-risk, high-yield diagnostic procedure such as lymphoscintigraphy with sentinel node biopsy for the accurate staging and management of CTCLs. Further research is certainly indicated to evaluate the rates of asymptomatic, nonradiographic evident node positive cutaneous malignancies. Furthermore the benefit of identifying and treating microscopic disease in nodes and the effect on prognosis should be addressed if trends in sentinel node biopsy as a staging procedure increase.
